# Gender differences in the effect of urge‐to‐cough and dyspnea on perception of pain in healthy adults

**DOI:** 10.14814/phy2.12126

**Published:** 2014-08-28

**Authors:** Peijun Gui, Takae Ebihara, Ryuhei Sato, Kumiko Ito, Masahiro Kohzuki, Satoru Ebihara

**Affiliations:** 1 Department of Rehabilitation Medicine, Toho University Graduate School of Medicine, Tokyo, Japan; 2 Department of Internal Medicine and Rehabilitation Science, Tohoku University Graduate School of Medicine, Sendai, Japan; 3 Department of International Oral Health, Tohoku University Graduate School of Dentistry, Sendai, Japan

**Keywords:** Analgesia, cough, gender, respiratory sensation

## Abstract

Previous studies have reported that respiratory sensations, such as urge‐to‐cough and dyspnea, have an inhibitory effect on pain. Considering the existence of gender differences in both urge‐to‐cough and pain, it is conceivable that a gender difference also exists in the analgesia induced by urge‐to‐cough. In this study, we evaluated gender differences in the pain perception response to urge‐to‐cough, as well as to dyspnea. Twenty‐seven male and 26 female healthy nonsmokers were originally enrolled. Citric acid challenge was used to induce the urge‐to‐cough sensation, and dyspnea was elicited by inspiratory loaded breathing. Before and during inductions of these two respiratory sensations, perception of pain was assessed by the thermal pain threshold, and differences between men and women were compared. The thermal pain threshold in women (43.83 ± 0.17°C) was significantly lower than that in men (44.75 ± 0.28°C; *P* < 0.05) during the baseline period. Accompanying increases in both citric acid concentration and inspiratory resistive load, thermal pain threshold values significantly increased in both men and women. The average thermal pain threshold changes for comparable increases in the urge‐to‐cough Borg score were parallel between men and women. Furthermore, the mean value of the thermal pain threshold plotted against the dyspnea Borg score also showed no significant gender difference. These results demonstrate that although gender differences exist in respiratory sensations, that is, urge‐to‐cough and dyspnea, the inhibitory effects of these respiratory sensations on the perception of pain are not significantly different between the sexes.

## Abstract (precis)

The relationships between citric acid dose and urge‐to‐cough rating or thermal pain threshold in each subject grouped by sex.

## Introduction

Bodily symptoms such as pain, cough, and dyspnea, are important responses to threatening and potentially harmful stimuli. They also frequently coexist in many clinical conditions, such as pleuritis, pulmonary embolism, and lung cancer. Although each of them has distinct features, they also share a number of similarities (Gracely et al. 2007). In clinical situations, these symptoms can be subjectively uncomfortable, signal bodily disturbances, and degrade the quality of life. Common therapies also suggest the analogies of these symptoms, for example, opioids, as characteristic analgesic drugs, show antitussive effect (Morice et al., 2007b) and alleviate certain types of dyspnea (Jennings et al. 2002). Brain functional magnetic resonance imaging studies have shown similar brain regions activated by pain, cough, and dyspnea (von Leupoldt et al. 2009; Mazzone et al. 2009). Considering these similarities, it is conceivable that interactions exist among the three symptoms.

Different from perceptions of pain and dyspnea, cough in humans is a motor action, usually associated with a characteristic sound (Morice et al., 2007a). Recent studies have shown that a respiratory sensation such as awareness of an irritating stimulus, named urge‐to‐cough, precedes the cough motor event (Davenport 2002; Mazzone et al. 2007). Moreover, the urge‐to‐cough sensation facilitates the cough response. Accompanied by enhanced perception of urge‐to‐cough, cough reflex sensitivity has been found to be greater in female subjects than in male subjects (Gui et al. 2010; Dicpinigaitis et al. 2012).

Gender differences in pain sensitivity and analgesia have also been widely reported, and compared with men, women are more likely to experience several clinical pain syndromes and tend to demonstrate increased pain sensitivity (Riley et al. 1998; Fillingim et al. 2004). The use of analgesics is much more frequent among women than men (Isacson and Bingefors 2002). Bearing in mind the gender differences in both urge‐to‐cough and pain sensitivities, it is possible to consider that there may be a gender difference in the interaction between urge‐to‐cough and pain. In the literature, however, little information is available concerning this. A recent study by Gui et al. showed that urge‐to‐cough, as well as dyspnea sensation, caused attenuation of pain sensation (Gui et al. 2012), but the study involved both male and female subjects and did not carefully take gender differences into account.

Hence, in this study, the gender differences in the response of the thermal pain threshold to urge‐to‐cough in healthy young subjects were evaluated. Furthermore, as a comparison, the gender differences in the response of the thermal pain threshold to dyspnea in healthy young subjects were examined. The hypothesis that both men and women might show inhibitory effects of respiratory sensations on the pain sensation was addressed, but which sex would have a greater inhibitory effect was not predicted.

## Materials and Methods

### Subjects

Healthy adult nonsmokers were enrolled to evaluate perception of urge‐to‐cough, dyspnea, and thermal pain. They were recruited via public posters in and around the campus. None of them showed clinical evidence of chronic pain, nor of cardiac, or respiratory disorders. Female subjects did not participate during menses, and none of them took any medicine. The Ethics Committee of Tohoku University School of Medicine approved this study (Approval number: 2010‐180‐1), and each subject signed an informed consent form prior to the start of the study.

### Citric acid challenge and assessment of urge‐to‐cough perception

Subjects underwent the citric acid cough challenge with an ultrasonic nebulizer (NE‐U17, Omron Co. Ltd., Kyoto, Japan) with measurement of the urge‐to‐cough intensity as previously described (Yamanda et al. 2008; Gui et al. 2010; Kanezaki et al. 2010). Briefly, subjects inhaled each citric acid aerosol dissolved in saline under tidal breathing for 1 min (Gui et al. 2010). The citric acid solutions were administered from 0.7 g/L to 360 g/L in increasing doubling concentrations (Yamanda et al. 2008). A placebo physiological saline aerosol was applied to the subjects first before inhaling the citric acid solutions. After each inhalation, 2‐minute rest intervals were applied between concentrations (Kanezaki et al. 2010). Following each inhalation, coughs were counted by an experienced operator. Subjects were told to cough freely and not to try to suppress coughing. The cough reflex threshold was defined as the lowest citric acid concentration that induced subjects to cough two or more times (C_2_) during a period of 1 min. The urge‐to‐cough intensity was evaluated according to a modified Borg scale ranging from 0 (no need to cough) to 10 (maximum urge‐to‐cough) (Davenport 2002) immediately after each citric acid inhalation (Table 1). The urge‐to‐cough threshold was defined as the initial concentration of citric acid which induced the urge‐to‐cough sensation without a preceding motor cough event and was termed C_u_ (Dicpinigaitis et al. 2012).

**Table 1.** Urge‐to‐cough Borg category scale in English and in Japanese.

Score	Urge‐to‐cough (English)	咳衝動 (Japanese)
0	No need to cough	まったく咳をしなくていい
0.5	Very, very slight	とてもとても軽い
1	Very slight	とても軽い
2	Slight	軽い
3	Moderate	中くらい
4	Somewhat severe	いくぶん咳をしたい
5	Severe	咳をしたい
6		
7	Very severe	大変咳をしたい
8		
9	Very, very severe (almost maximal)	大変大変咳をしたい
10	Maximum urge‐to‐cough	最大限咳をしたい

The urge‐to‐cough Borg scale used by Davenport PW et al. was translated from English to Japanese by one of the authors (SE).

### Perception of dyspnea

Dyspnea sensation was induced by inspiratory loaded breathing and measured according to the modified Borg scale (Kikuchi et al. 1994; Ebihara et al. 2002; Gui et al. 2010; Kanezaki et al. 2010). In brief, the subject breathed through the previously described apparatus using a unidirectional Hans‐Rudolph valve with linear inspiratory resistances (R) of 0, 10, 20 and 30 cmH_2_O/L/s (Kikuchi et al. 1994; Ebihara et al. 2002). Progressively increased resistances were applied to each subject. The subjects were not told to control either the ventilation or breathing pattern during the test. After breathing for 1 min at each load, the subjects were shown a modified Borg scale, which they used for rating, indicating whether they had a dyspnea sensation (breathing discomfort), and if so, its intensity (0, no dyspnea; 10, maximal dyspnea) (Kikuchi et al. 1994) (Table 2). In practical situations, before the measurement, we told each subject to rate the sensation of kokyu‐konnan or “discomfort of breathing” while breathing with resistance (Gui et al. 2010). The term kokyu‐konnan is an exact Japanese translation of dyspnea. Kokyu means breathing or respiration, and konnan means discomfort or difficulty (Gui et al. 2010). The term kokyu‐konnan was not defined any further. The Borg score at every load and the summation of all loads were compared between sexes.

**Table 2.** Dyspnea Borg category scale in English and in Japanese.

Score	Dyspnea (English)	呼吸困難 (Japanese)
0	No dyspnea	全然なし
0.5	Very, very slight	とてもとても軽い
1	Very slight	とても軽い
2	Slight	軽い
3	Moderate	中くらい
4	Somewhat severe	いくぶんきつい
5	Severe	きつい
6		
7	Very severe	大変きつい
8		
9	Very, very severe (almost maximal)	大変大変きつい
10	Maximum dyspnea	最大

The dyspnea Borg scale used by Kikuchi Y et al. was translated from English to Japanese by one of the authors (SE).

### Thermal pain sensitivity

To assess the thermal pain threshold (TPTh), a contact thermode 1 cm in diameter (UDH‐105, Unique Medical Co. Ltd., Japan) was placed on the left volar forearm (Gui et al. 2012). The temperature of the thermode was increased from 35°C at a rate of 0.25°C/s until the thermal percept first became painful (i.e., the lowest painful temperature). For safety reasons, temperatures never exceeded 51°C (Gui et al. 2012). In order to avoid peripheral receptor sensitization or habituation, the thermode was shifted to a different, but adjacent position on the volar forearm between trials.

### Experimental protocol

Testing was performed on three different days within 1 week as previously described (Gui et al. 2012). For logistical reasons, the day intervals could not be equivalent for all subjects. A short simple standard instruction was given to accustom subjects to the apparatus and the use of the modified Borg scale. On the first study day, subjects performed spirometry first. Then, thermal pain sensation, dyspnea perception, cough reflex, and urge‐to‐cough were examined according to the above‐described criteria (Gui et al. 2012). A previous study found that day‐to‐day variation was slight for thermal pain perception (Agostinho et al. 2009). A 5‐min interval was applied for each measurement.

On the subsequent two study days, the thermal pain threshold was assessed in each subject according to randomly selected experimental conditions, that is, during inspiratory loaded breathing and during citric acid inhalation. In one condition, the thermal pain threshold was assessed under inspiratory loaded breathing. The subjects breathed through the Hans‐Rudolph valve with linear inspiratory resistances. The magnitude of inspiratory resistance (R) was 0, 10, 20, and 30 cmH_2_O/L/s. The duration of each load was 1 min, and the order of inspiratory resistance (R) was randomized. In the other condition, subjects were requested to inhale a physiological saline or citric acid aerosol randomly. During the inhalation, a noxious heat stimulus was applied, and the thermal pain threshold was estimated. To induce the urge‐to‐cough sensation without the motor act, the citric acid concentrations of eight times, four times, and two times dilution of C_2_ (C_2_/8, C_2_/4, C_2_/2) were applied, based on the citric acid challenge results from the first study day (Mazzone et al. 2007; Yamanda et al. 2008; Gui et al. 2012).

### Data analysis

Data are expressed as mean ± SEM except where otherwise specified. The Mann–Whitney *U* test was used to compare male and female variables. The effects of citric acid concentration and gender on intensity of urge‐to‐cough and thermal pain threshold were assessed using two‐way repeated measures analysis of variance followed by the Bonferroni post hoc test. The effects of inspiratory loading and gender on intensity of dyspnea and thermal pain threshold were also evaluated using the same analysis. Pearson's correlation coefficient was used to evaluate correlations between variables. A *P* value of <0.05 was considered significant.

## Results

Fifty‐three subjects (mean age 28.25 ± 7.15 (SD) years; 27 male, mean age 28.85 ± 5.31 years; 26 female, mean age 27.62 ± 8.73 years) were enrolled in and completed all the experimental protocols without any difficulty or side effects. Subject characteristics are summarized in Table 3. Age, spirometric measures expressed as percent predicted, and the FEV_1_/FVC ratio revealed no significant gender differences. In terms of gender comparison, the cough reflex threshold, as expressed by log C_2_, was significantly lower in women (0.99 ± 0.27 g/L) than in men (1.24 ± 0.37 g/L; *P* < 0.03), indicating greater enhanced cough reflex sensitivity in female subjects than in male subjects. The citric acid concentrations of eight times, four times, and two times dilution of C_2_ (C_2_/8, C_2_/4, C_2_/2), as expressed by log transformation, were significantly lower in women than in men. However, no statistically significant gender difference was found for the urge‐to‐cough threshold log C_u_.

**Table 3.** Comparison of characteristics between males and females.

Characteristic	Male	Female	*P* value^1^
No.	27	26	
Age, y	28.85 ± 5.31	27.62 ± 8.73	0.06
FEV_1_, % predicted	102.27 ± 10.19	100.41 ± 15.76	0.63
FVC, % predicted	111.38 ± 14.00	109.25 ± 17.50	0.87
FEV_1_/FVC ratio, %	87.59 ± 5.67	90.70 ± 5.80	0.05
PEF, % predicted	94.19 ± 17.03	89.06 ± 18.39	0.44
Cough response			
Log C_2_ (g/L)	1.24 ± 0.37	0.99 ± 0.27	0.01
Log C_2_/2 (g/L)	0.91 ± 0.35	0.65 ± 0.30	0.01
Log C_2_/4 (g/L)	0.60 ± 0.35	0.36 ± 0.26	0.01
Log C_2_/8 (g/L)	0.30 ± 0.35	0.07 ± 0.24	0.01
Log C_u_ (g/L)	0.35 ± 0.45	0.39 ± 0.24	0.24
Dyspnea response			
Dyspnea Borg sum	7.54 ± 3.79	10.04 ± 3.94	0.04

Data are presented as mean ± SD, unless otherwise indicated. PEF = peak expiratory flow.

^1^ *P* values were calculated by the Mann–Whitney *U* test.

**Figure 1.**
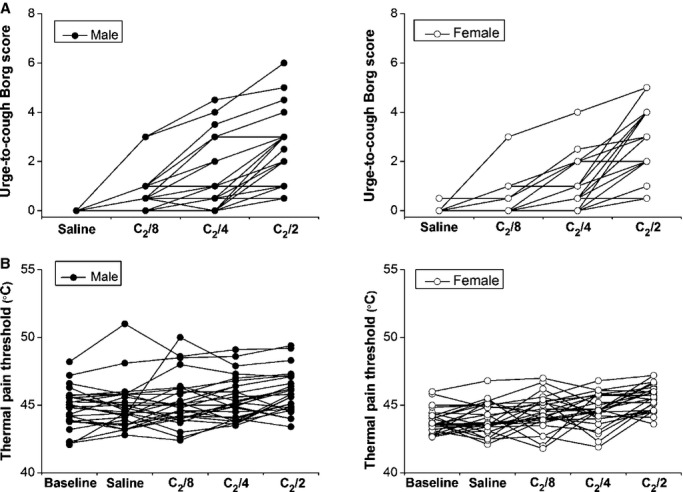
The relationships between citric acid dose and urge‐to‐cough rating (A) or thermal pain threshold (B) in each subject grouped by sex.

**Figure 2.**
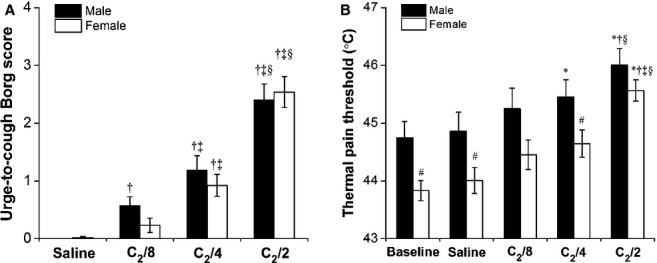
Mean values of urge‐to‐cough Borg scores (A), thermal pain threshold (B) during urge‐to‐cough in the response to citric acid challenge between men (closed bars) and women (open bars). Symbol (*) indicated a significant difference (*P* < 0.05) compared with values at baseline; (†) indicated a significant difference (*P* < 0.05) compared with values during physiological saline inhalation; (‡) indicated a significant difference (*P* < 0.05) compared with values during inhalation of eight times dilution of C_2_ citric acid solution; (§) indicated a significant difference (*P* < 0.05) compared with values during inhalation of four times dilution of C_2_ citric acid solution; (#) indicated a significant difference (*P* < 0.05) compared with male for the corresponding citric acid concentration.

Figure 1 shows the relationship between citric acid dose and urge‐to‐cough rating (Fig. 1A) or thermal pain threshold (Fig. 1B) between sexes for individuals. As demonstrated in Figure 2A, urge‐to‐cough scores for physiological saline and for the concentrations of eight times, four times, and two times dilution of C_2_ (C_2_/8, C_2_/4, C_2_/2) were evaluated among male and female subjects. Figure 2B shows the gender difference in thermal pain threshold as a function of the urge‐to‐cough sensation. At the baseline, the thermal pain threshold was significantly lower in women (43.83 ± 0.17°C) than in men (44.75 ± 0.28°C; *P* < 0.01). Accompanying an increase in the citric acid concentration, the value of the thermal pain threshold significantly increased during induction of the urge‐to‐cough sensation in both men and women. The results of the two‐way repeated ANOVA indicated that a main effect of citric acid concentration on thermal pain threshold (*F* = 23.68, *P* < 0.001). There was a significant effect of gender on thermal pain threshold (*F* = 5.88, *P* < 0.02). There was no significant interaction between the effect of citric acid concentration and gender (*F* = 0.60, *P* = 0.64). The gender difference in thermal pain threshold remained significant during physiological saline inhalation (*P* < 0.05), whereas no significant gender difference in this value was observed during inhalation of two times dilution of C_2_ citric acid solution.

**Figure 3.**
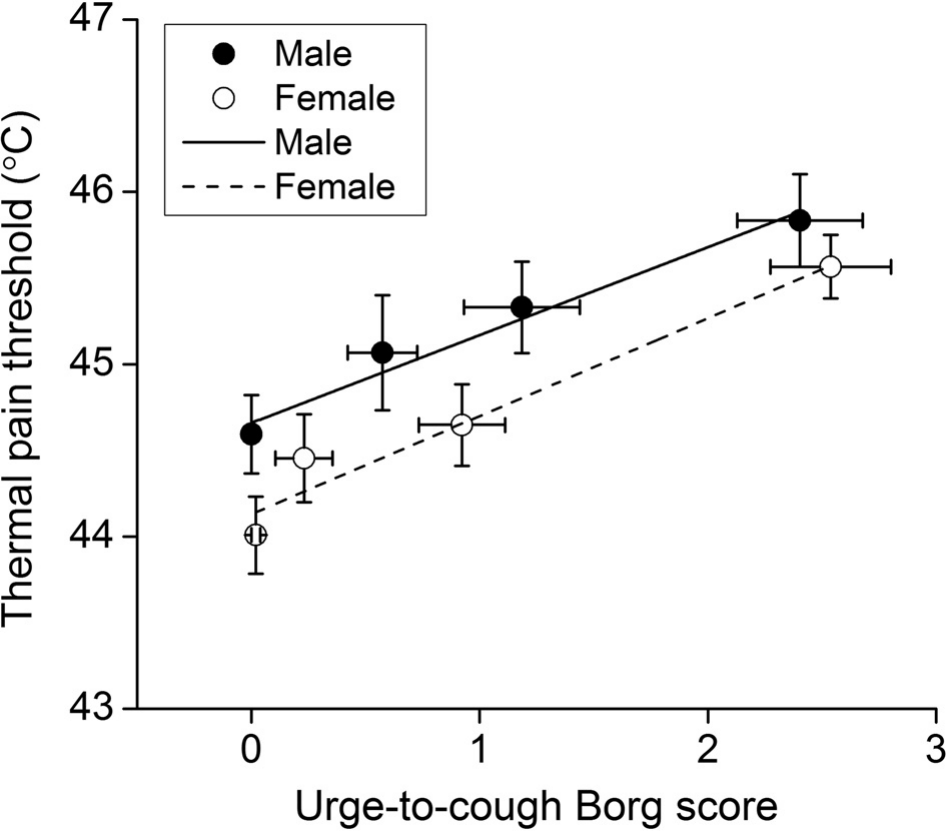
Increases in thermal pain threshold as a function of urge‐to‐cough induced by citric acid challenge between men (solid circles) and women (open circles). Lines are corresponding mean regression slopes. The slopes of the response curves for men and women were 0.51 and 0.57, respectively.

Figure 3 shows the average thermal pain threshold changes for comparable increases in the urge‐to‐cough Borg score as a function of citric acid concentrations in men and women. The mean slopes of the response curves for men and women were 0.51 and 0.57, respectively. This suggests that the changes in thermal pain threshold at comparable levels of the urge‐to‐cough Borg score were parallel between men and women, indicative of the absence of a significant gender difference in inhibitory rate of pain perception by urge‐to‐cough.

**Figure 4.**
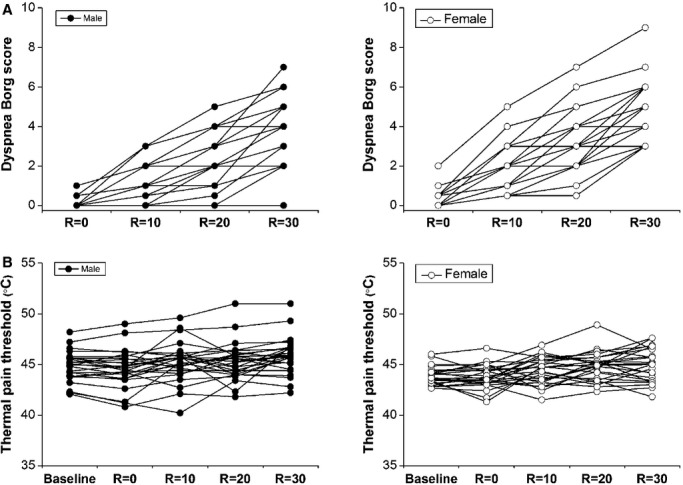
The relationships between inspiratory resistive load and dyspnea Borg scores (A) or thermal pain threshold (B) in individual subjects grouped by sex.

**Figure 5.**
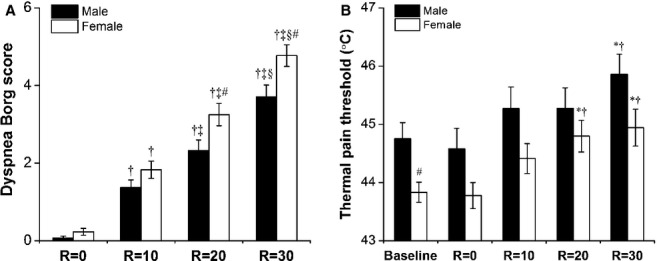
Mean values of dyspnea Borg scores (A), thermal pain threshold (B) during breathing discomfort in response to inspiratory resistive load at *R* = 0, 10, 20, 30 cmH_2_O/L/s between men (closed bars) and women (open bars). Symbol (*) indicated a significant difference (*P* < 0.05) compared with values at baseline; (†) indicated a significant difference (*P* < 0.05) compared with values during *R* = 0 cmH_2_O/L/s resistive loading; (‡) indicated a significant difference (*P* < 0.05) compared with values during *R* = 10 cmH_2_O/L/s resistive loading; (§) indicated a significant difference (*P* < 0.05) compared with values during *R* = 20 cmH_2_O/L/s resistive loading; (#) indicated a significant difference (*P* < 0.05) compared with male for the corresponding resistive loading.

Similarly, Figure 4 shows the relationship between inspiratory resistive load and dyspnea Borg scores (Fig. 4A) or thermal pain threshold (Fig. 4B) in individual subjects. Figure 5A shows the intensities of perception of dyspnea during the external inspiratory resistive loads between men and women. There were no significant gender differences in the Borg scores at *R* = 0 and 10 cm H_2_O/L/s (*P* = 0.12 and 0.13, respectively). However, the Borg scores at *R* = 20 and 30 cm H_2_O/L/s were significantly less in men than in women (*P* = 0.03 and 0.01, respectively). Accompanying an increase in the inspiratory resistive load, the thermal pain threshold significantly increased both in men and in women (Fig. 5B). There was a significant main effect of resistive load on thermal pain threshold (*F* = 16.95, *P* < 0.001). A significant effect of gender on thermal pain threshold was also observed (*F* = 4.72, *P* < 0.04). There was no significant interaction between the effect of resistive load and gender (*F* = 0.54, *P* = 0.67).

**Figure 6.**
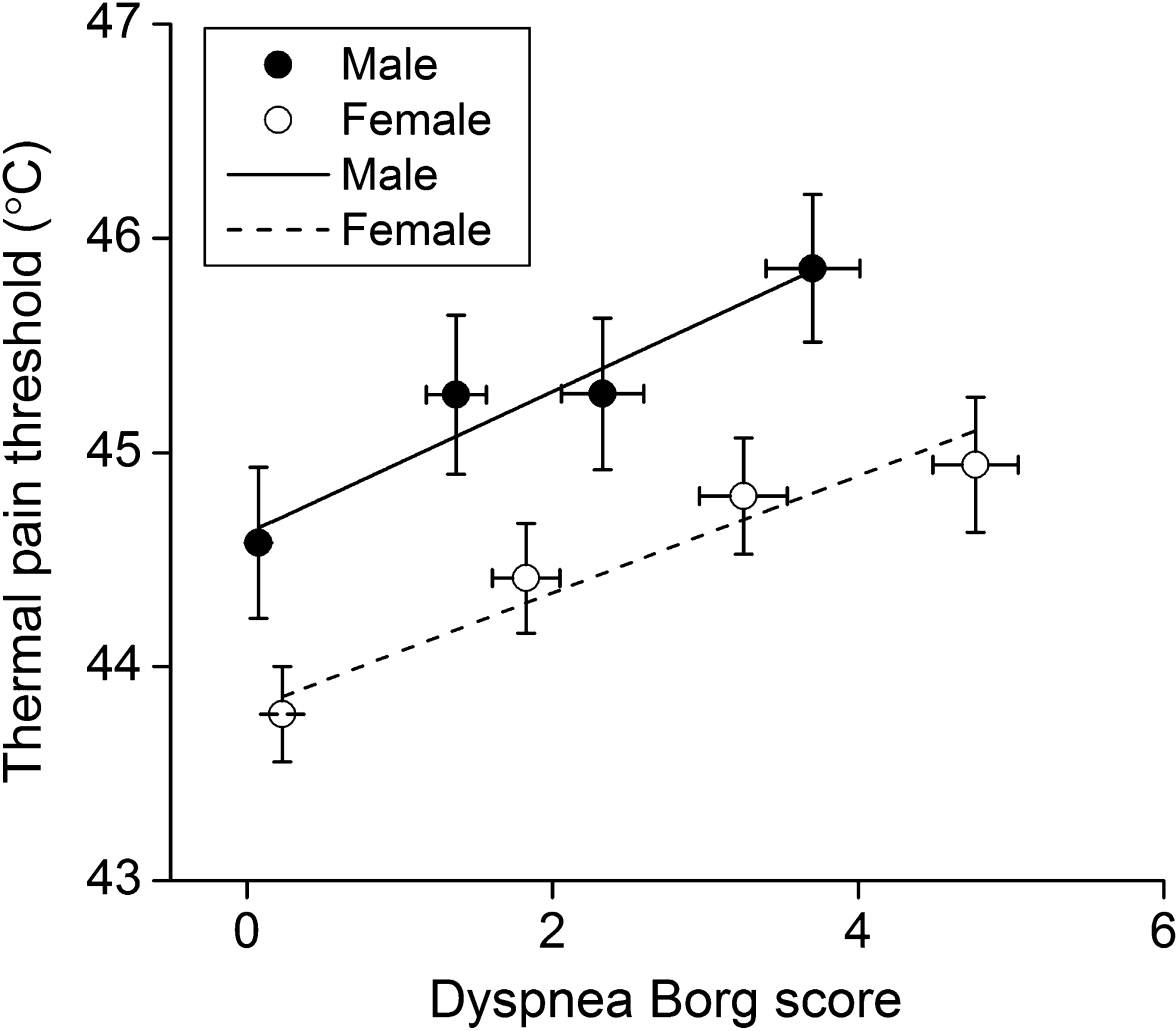
Average values of thermal pain threshold plotted against dyspnea Borg score in response to inspiratory resistive load at *R* = 0, 10, 20, 30 cmH_2_O/L/s between men (solid circles) and women (open circles). Lines are corresponding mean regression slopes. The slopes for men and women were 0.33 and 0.27, respectively.

The mean value of the thermal pain threshold plotted against the dyspnea Borg score induced by inspiratory resistive loads is shown in Figure 6. In terms of the gender comparison, parallel changes were found in the thermal pain threshold as a function of the dyspnea Borg score between men and women. The average slopes of the response curves for men and women were 0.33 and 0.27, respectively. It suggests that no gender difference existed in the inhibitory rate of pain perception by dyspnea.

## Discussion

In this study, we first demonstrated greater thermal pain sensitivity among women than men at baseline, which is consistent with previously published studies (Riley et al. 1998; Fillingim et al. 2004). Then, accompanying increases in both citric acid concentration and inspiratory loading, thermal pain thresholds significantly increased in both men and women. Moreover, although gender differences existed in the perception of both urge‐to‐cough and dyspnea, the inhibitory rates of pain perception by both urge‐to‐cough and dyspnea did not differ between men and women.

The results are consistent with previously published reports, which have reported analgesic effects of urge‐to‐cough (Gui et al. 2012) and dyspnea (Morelot‐Panzini et al. 2007; Nishino et al. 2008; Bouvier et al. 2012). However, the mechanism for this analgesic phenomenon has not been fully explored. Morélot‐Panzini et al. suggested that dyspnea may trigger subcortical endogenous analgesia (Morelot‐Panzini et al. 2007), whereas other studies have indicated that respiratory sensations induced endogenous analgesia may also occur at the cortical level. Investigation of gender differences in analgesia induced by respiratory sensations as in this study may contribute to the understanding of the analgesic mechanism.

Gender differences in analgesia have been investigated using clinical and experimental pain models. No sex difference was found in morphine analgesia for acute pain in the emergency department (Bijur et al. 2008). Also, the majority of experimental studies have reported no sex difference in analgesia with opioids, such as morphine (Fillingim et al. 2005) and pentazocine (Fillingim et al. 2004). In addition to the above findings addressing opioid analgesia, other studies have reported that a gender difference exists in analgesic response to nonsteroidal anti‐inflammatory drugs (NSAIDs), cholinergics, cannabinoids, anesthetics, and also nonpharmacologic interventions. In view of the known gender differences in the effect of analgesic drugs (Fillingim et al. 2009), various biological and psychosocial factors likely contribute to gender differences in analgesia.

Urge‐to‐cough, as a respiratory sensation preceding a cough, is a component of the brain motivation system mediating cognitive responses to cough stimuli (Davenport 2008). It is the result of a combination of peripheral afferent inputs from peripheral sensors and modulation of the central nervous system (Gracely et al. 2007). Peripheral sensors, such as the A‐δ nociceptors, cough receptors, pulmonary, and bronchial C‐fibers are thought to be associated with induction of the urge‐to‐cough sensation (Widdicombe 2009). Brain functional magnetic resonance imaging studies have shown that urge‐to‐cough activated a variety of brain regions, such as the insular cortex, the anterior cingulate cortex, and the cerebellum (Mazzone et al. 2007; Leech et al. 2013), which were also activated by noxious thermal pain perception (Farrell et al. 2005). Although urge‐to‐cough is evoked by airway sensory nerve stimulation, and pain perception is evoked by peripheral somatosensory stimulation, parts of the same brain regions are activated by both sensations (Mazzone et al. 2009). Since urge‐to‐cough induced an analgesic effect in this study, it is possible that the inhibitory effect of urge‐to‐cough on pain perception may be due to the shared brain region activated by urge‐to‐cough. Furthermore, the results demonstrated that although women showed higher perception of urge‐to‐cough and pain, there was no significant gender difference in the inhibitory rates of pain perception by urge‐to‐cough (Fig. 3), suggesting that gender difference may not be obvious in central processing of analgesia.

In addition, dyspnea, as another respiratory sensation, also showed an inhibitory effect on pain perception. The ATS Official Statement on dyspnea has reported that dyspnea comprises several different uncomfortable respiratory sensations like work/effort, air hunger, tightness (Parshall et al. 2012). These distinct sensations most often do not occur in isolation (Parshall et al. 2012). According to our previous study, dyspnea, which was induced by inspiratory resistive loading with the identical protocol as in this study, comprised both work/effort and air hunger sensations (Kashiwazaki et al. 2013). Spinal nociceptive flexion reflex inhibition only occurred when dyspnea of the “work/effort” type but not “air hunger” type was induced (Morelot‐Panzini et al. 2007). On the other hand, a recent study by Yashiro et al. (2011). showed that both air hunger and work/effort caused a similar degree of pain inhibition induced by cold‐pressor test. The result was consistent with this study.

However, there was no significant gender difference in response to dyspnea‐induced analgesia (Fig. 6). Previous studies have reported that dyspnea induced by inspiratory resistive loading may activate the endogenous opioid system in an intensity‐dependent manner (Scardella et al. 1986; Gifford et al. 2011). The *β*‐endorphin level in the cerebrospinal fluid (CSF) was increased after inspiratory resistive loading (Scardella et al. 1986). Many studies have reported no sex difference in analgesia with a variety of opioids (Fillingim et al. 2004, 2005). The endogenous opioid system may play a role in the gender difference of dyspnea‐induced analgesia in this study. Also, considering the similarities between dyspnea and urge‐to‐cough, the gender difference of urge‐to‐cough induced analgesia may be due to the modulation of the endogenous opioid system. Future research using brain imaging to observe the effect of respiratory sensation on pain perception is necessary to clarify the role of gender related to pain perception.

In conclusion, despite the existence of gender differences in respiratory sensations, that is, urge‐to‐cough and dyspnea, the inhibitory effects of these respiratory sensations on the perception of pain are not sex‐specific. Modulation of the endogenous opioid system is possibly involved in this gender difference. Regardless of gender, when it is difficult to breathe or when it is necessary to cough to remove airway irritations, perhaps pain does not matter. The findings bring new elements to the physiological understanding of the nature of the interaction between respiratory sensations and pain.

## Conflict of Interest

None declared.
